# Magnetic Sensing Properties of PVD Carbon Films Containing Vertically Aligned Crystallites

**DOI:** 10.3390/s19194248

**Published:** 2019-09-30

**Authors:** Xingze Dai, Jing Guo, Tongbin Huang, Dong Ding, Chao Wang

**Affiliations:** Institute of Nanosurfacce Science and Engineering, College of Mechatronics and Control Engineering, Shenzhen University, Shenzhen 518060, China; 1800291007@email.szu.edu.cn (X.D.); guojing2017@email.szu.edu.cn (J.G.); 2172291716@email.szu.edu.cn (T.H.); dingdong721@szu.edu.cn (D.D.)

**Keywords:** magnetic sensor, room temperature, physical vapor deposition, nano-crystallited carbon film, anisotropy, substrate enhancement

## Abstract

The demands for magnetic sensors are uprising due to the rapid development of smart equipments and internet of things. Exploring magnetic sensitive materials which are easily obtainable and of low cost thereby become of great significance. Carbon film with crystallized features was recently reported with room-temperature ferro-magnetism and magnetoresistance, owing to its spin–orbital interactions at the graphene edges and temperature-depending carrier transport properties. Such phenomena indicate that the film can serve as a novel magnetic sensitive material. In this study, carbon films with vertically aligned nano-crystallites were obtained by a plasma-assisted physical vapor deposition (PVD) method. Basic test circuits were built on the films, and the sensing properties were investigated in external magnetic fields with different intensities and relative angles to the films surface. The results showed that the carbon-based sensing devices were capable to work in the temperature region of 250–400 K. The minimum field intensity and angle change to which the device can respond were 1 mT and 2°. By substrate-introduced enhancement, the maximum changing-rate of the film resistance could reach to 1100%/T. This research pointed out a practical and simple way to build magnetic sensors with PVD carbon films.

## 1. Introduction

Carbon films have been widely utilized in the fields of film sensor as either sensitive devices [1,2,3,4,5] or protective layer [6]. Through detecting the electric response of carbon films to certain input signal, they are designed as strain/stress sensor [1,2], biological sensor [3,4], and electrochemical sensors [4,5]. With the discovery of graphene in the laboratory, due to its distinctive electrical, photonic, mechanical properties, carbon-based sensors are promoted to the scale of nano-size and few atomic layers. In addition, few layer graphene/graphene nano ribbons have been reported to be used as biosensors [7,8,9], stress sensor [10,11], and color sensor [12]. However, the difficulty of the massive fabrication of graphene devices impeded the further development and practical application of such sensors [13,14,15].

Nano-crystallited carbon film has gained much attention due to its excellent electrical [16,17], magnetic [18,19], and tribological [20,21] properties, especially its magnetoresistance (MR) at room temperature [22]. The novel electrical response of this film under external magnetic field shed light on a new-type of carbon-based magnetic sensing device. In a recent study, it has been found that the RT-MR performance of graphene nano-crystallited film is originated from its self-magnetism enhancement and was highly related to the content of itinerant electrons in the film [22]. This phenomenon provided a new possibility to further improve the RT-MR properties by increasing the density of itinerant electrons. However, potential magnetic sensing ability of the nano-crystallited carbon film was overlooked, and the efforts to improve its property was insufficient, which is of great significance for the practical applications of carbon-based film magnetic sensor.

In this study, n-type silicon wafer was used as the “donor” substrate, and nano-crystallited carbon film was grown on the substrate by electron irradiation assisted physical vapor deposition (PVD) in electron cyclotron resonance (ECR) plasma. The graphene nano-crystallites in the film matrix were identified with high resolution transmission electron microscopy (TEM) and Raman spectra. Film surface morphology was measured with atomic force microscopy (AFM). The magnetic sensing device based on C-film/n-Si was built with a four-probe method, and its magnetic sensing performances including magnetoresistance (MR) and anisotropic magnetoresistance (AMR), the sensitivity and resolution, and their temperature dependence were investigated by using the physical property measurement system (PPMS). A reference-device from C-film/SiO_2_ was built and tested for comparison, and the enhancement of the n-Si substrate on MR performance of the nano-crystallited carbon film was discussed. The potential of magnetic sensing ability of the carbon film and the possible route to improve the current device were prospected based on the discussion.

## 2. Materials and Methods

### 2.1. Film Deposition and Test Sample Preparation

Phosphor-doped silicon wafers (n-type, electrical resistivity 20–30 Ω·cm) with the thickness of 500 μm was used as substrates for film deposition. The carbon film was deposited in an ECR plasma electron/ion irradiation system [17,23], which is capable of film deposition at the scale of 2-inch-radius at maximum. In this research, the wafer was cut into 25 × 25 mm^2^ pieces, then pre-treated with acetone ultrasonic cleaning before inserting into vacuum chamber. A base pressure of 10^−5^ Pa was achieved in the chamber before argon was introduced to generate high density plasma, and a negative substrate bias of −50 V was applied to realize Ar^+^ ion irradiation to remove the oxide layer on top surface of the substrate, thus obtaining good contact between the film and the substrate. A cylinder target consisting of glassy carbon was used to provide the carbon atoms for film growth. The nano-crystallited carbon film was deposited under electron irradiation with the energy of 100 eV, which as modulated by positive substrate bias [23]. Electrodes were fabricated on the film surface with silver paste and Pt wires in four-electrode configuration. The electrodes were parallel aligned in order to as much avoid the effect of Hall voltage, which could result in different magneto-voltages between center electrodes in opposite directions of magnetic field.

### 2.2. Film Characterization and Electric Measurement

The thickness of the film was controlled by deposition time. The film thickness was measured from the side-view of the film-substrate interface in TEM observations. The fine structure of the nano-crystallites was observed in the top-view TEM. The side-view TEM sample was prepared by dual-beam focus ion beam etching method. For top-view TEM sample preparation, the film was scratched off the substrate by a diamond pencil, which can peel off the film without carving too deep into silicon substrate. The flakes of the film were transferred onto lacey carbon grid by sliding the grid on scratched debris. TEM observation was performed in Titan Cubed Themis G2 microscope under the acceleration voltage of 80 keV to pretend the carbon film from graphitization or being damaged too fast under high voltage electron beam. The binding configurations of the films were analyzed by Raman spectra, which were obtained with Horiba HR Evolution spectrometer using a 532 nm laser for excitation with the power of <1 mW and spot size of 1 μm. Each spectrum was acquired in less than 5 min in case the film structure was changed by laser irradiation. The surface roughness was measured with ASIT SPM-1000 microscope in tapping mode. The MR and AMR measurements were carried out in a Quantum Design Dynacool system in the magnetic field range of 0–9 T, and temperature range of 2–400 K. The MR value was defined as *Δρ* = (*R* − *R*_0_)/*R*_0_, where *R*_0_ is the electrical resistance in zero field, and *R* is the resistance in a non-zero external magnetic field. AMR value was defined as *Δρ* = (*R_θ_* − *R*_0°_)/*R*_0°_, where *R*_0°_ is the resistance when film surface was in parallel to the magnetic lines, and *R_θ_* is the resistance when film surface was in the angle of *θ* with respect to the magnetic lines.

## 3. Results and Discussion

### 3.1. Characterization of the Nano-Crystallited Carbon Film

**Figure 1:**
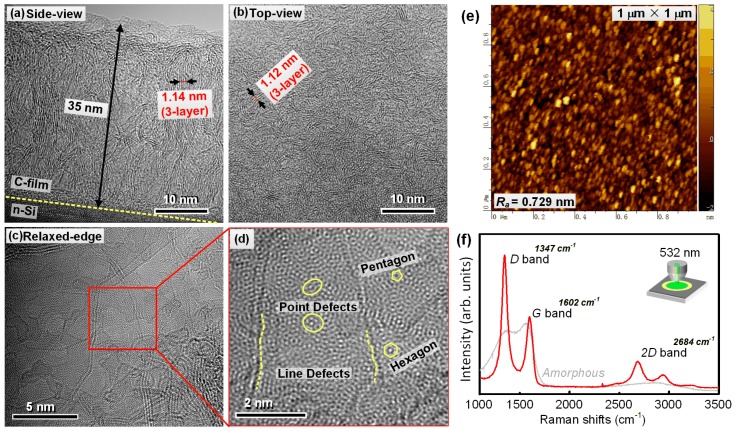
(**a**) Side-view TEM image of the nano-crystallited carbon film. The interface between film and substrate was indicated with yellow dash line. The distance between the atomic layers was indicated with arrows; (**b**) top-view TEM image of the nano-crystallited carbon film. The distance between the atomic layers was indicated with arrows; (**c**) high resolution TEM images at the relaxed-edge site of the film where overlapped atomic layers can be identified; (**d**) magnified image of the red-square range in (**c**). The Moiré pattern formed from honeycomb-shaped graphene lattice and point/line defects are well illuminated; (**e**) atomic force microscopy (AFM) surface morphology image of the carbon film within 1 × 1 μm^2^ area, and the *Ra* roughness of 0.729 nm was obtained; (**f**) Raman spectrum of the carbon film, where *D*, *G*, and *2D* bands were labeled with their peak positions. The spectrum of amorphous carbon film was also exhibited in grey color for comparison.

The characterization results including TEM images, AFM image, and Raman spectrum were shown in Figure 1. From the side-view TEM image in Figure 1a, the thickness of the film was clearly revealed as about 35 nm. The crystallites can also be identified inside the film matrix, which aligned approximately normal to the Si surface, in other words, vertical to the growing direction of the film. In order to investigate the phase of the crystallites, the spacing of the atomic layers was measured, as indicated with arrows in the image. Since the TEM images here are bright field images, the atomic layers should appear as black lines. In Figure 1a, the value of 1.14 nm was obtained across four black lines (thin red line), including three inter-layer distances, which equals to 0.38 nm between adjacent atomic layers, suggesting a graphitic phase. Such spacing is a little larger than the distance between graphene layers (0.345 nm). This may be due to the defect-rich feature of the crystallites, which can lead to twisting and expanding of the graphene lattice along inter-layer direction. Figure 1b showed the top-view TEM image of the film. The image exhibited rich amount of short-range ordered nano structure. The spacing between atomic layers were investigated with the same method as in side-view image, and the value of 1.12 nm was obtained, which equals to 0.37 nm, similar to the side-view image. This result suggested that the nano-sized crystallites in the film are graphene crystallites. Not as in side-view image, the orientation of the graphene layers is random, suggesting the film is only anisotropic along thickness direction, and isotropic along surface-plane direction. During TEM observation, a very thin region of the film flake was found (Figure 1c), where the graphene sheets were already unfolded due to the release of inner stress. At this relaxed-edge, the orientation of the graphene sheets was different from their original grown direction inside the film matrix. On the other hand, the few-layer area gave a much clearer projection of the graphene crystallites, where the honeycomb arrangement of carbon atoms can be identified. Meanwhile, the Moiré patterns from multilayer graphene and abundant amount of point and line defects were vividly presented, as the markers indicate in Figure 1d, zooming from Figure 1c.

The surface morphology of the film was shown in Figure 1e, which consisted of densely distributed clusters. The sizes of the clusters were around 5–10 nm according to the AFM image, and the *R_a_* roughness of the measured surface area was 0.729 nm. Such morphology implied that the film growth was in the form of many small islands which developed separately during deposition. Combing with TEM images, since there were no clear boundaries between the nano-sized crystallites, it can be inferred that the crystallites were somehow connected with each other. This feature is beneficial for the electric conduction of the film as a sensing device.

The Raman spectrum of the nano-crystallited film is shown in Figure 1f in which the spectrum of amorphous carbon film is given for comparison. For nano-crystallited film, the typical *D* and *G* bands with the position of 1347 cm^−1^ and 1602 cm^−1^ were marked in the Figure 1f. The above positions were obtained by fitting the spectrum region of 1100–1800 cm^−1^ with Lorentz and Breit-Winger-Fano methods [18]. On the contrary, the amorphous film possessed a less-separate band pack, in which the *D* and *G* band merged together due to their large band width. Moreover, the spectrum of nano-crystallited film showed a *2D* band in the position of 2684 cm^−1^, normally representing the existence of few-layer graphite or graphene. This feature coincided with the TEM observation results. On the contrary, the amorphous film did no show *2D* peak in similar region. As in our previous study, the appearance of *2D* band represented the formation of graphene nano-crystallites. [16] Therefore, the *2D* band in Figure 1f also confirmed the nano-crystallited structure of the carbon film.

### 3.2. Magnetic Sensing Performances in Magnetic Field with Different Intensities

**Figure 2:**
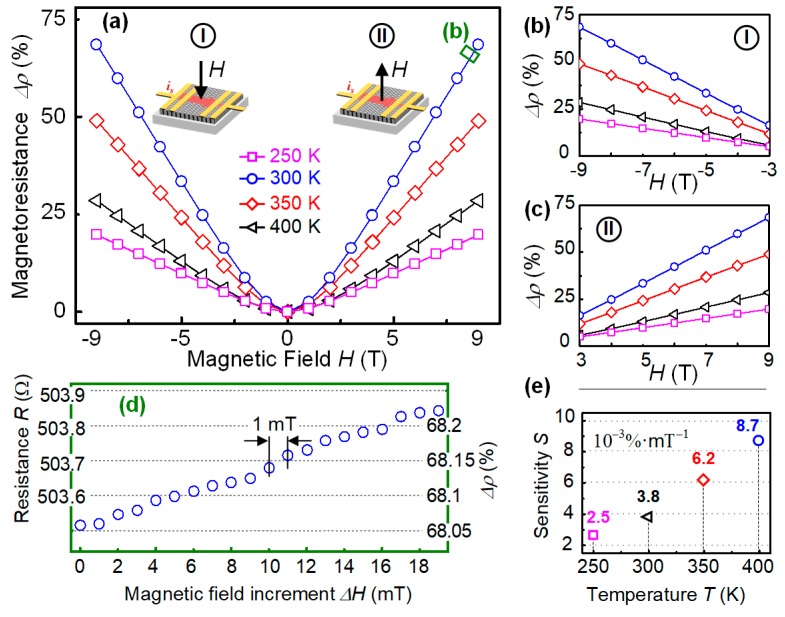
Magnetic sensing property of carbon film in perpendicular external magnetic field. (**a**) Temperature dependent magnetoresistance from 250–400 K. The intensity of the magnetic field was increasing from zero to 9 T along opposite directions as illustrated inside the figure; (**b**,**c**) linear magnetoresistance of the carbon film in high intensity field range |H| of 3–9 T, and the temperature dependency; (**d**) resistance and magnetoresistance in the magnetic field range of 8.946–8.966 T (indicated with green square in (**a**)), with increment of 1 mT at 300 K, implying that the finest resolution can reach 1 mT; (**e**) calculated sensitivity at different temperatures from (**b**,**c**).

The magnetic sensing device was built by using nano-crystallited carbon film, and its sensing performances responding to external field with increasing intensities were investigated in the temperature region of 250–400 K, crossing the room temperature range for practical applications. The direction of the magnetic field was normal to the film surface, as illustrated in Figure 2a, with opposite direction in the measurement periods of I and II. The results showed that the devices presented positive magnetoresistance (MR) in responding to the external field from 0 to ±9 T. As temperature increased from 250 to 400 K, the largest MR changing ratio appeared at 300 K. This is due to the largest inner magnetization of the nano-crystallited structure at this temperature [23], leading to the strongest magnetic-field influence on the transport of the carriers. In the field range of 3~9 T and −9~−3 T, the device showed linear sensing performances, as indicated in Figure 2b,c, respectively, where the increase of the slope with temperature can be better identified. In order to estimate the best sensing ability of the device to minor field change, we chose the strong field range at high temperature for evaluation, which was from 8.946 to 8.966 T at 300 K, with the increment of 1 mT. Figure 2d exhibited the plots of the absolute resistance value of the device in each step of increasing field intensity and their corresponding MR value. Apparently, the reading of the data showed a monotonic increase as field intensity increased, and the increment between each step was about 0.02 Ω, which was easy to detect for the measurement equipment. It should be noted that the MR increment of each step was not exactly the same, leading to the distortion from perfect linear trend. This may come from the fluctuation of the magnetic field during measurement, and the limited precision of the test circuit, including geometric error of the sample and manual wiring. Nevertheless, we can still conclude that at current circumstance, the best sensing resolution of the device can reach to 1 mT. Such resolution should be expected in wider range of field intensities and temperatures if the quality of the device were improved.

We summarized the temperature dependence of the sensitivities in the linear responding range of 3–9 T, and the results are shown in Figure 2e. It can be seen that from 250 to 400 K, the sensitivity varied from 0.0025% to 0.0087% per mT. The ratio between the two sensitivities was over three times, indicating that the device can provide better sensitivity in room temperature, and device calibration at different temperature is indispensable for real application. This means, before practical usage as magnetic sensor, the zero-field resistance of the device in different temperature should be obtained as a database. When the device is working, the temperature of the working environment should be given at the same time from another detector or checking the zero-field resistance of the device, then a correct magnetic field intensity can be obtained by measuring the in-field resistance of the device. If such conditions are met, the magnetic sensor based on the nano-crystallited carbon film can be realized.

### 3.3. Magnetic Sensing Performances in Magnetic Field with Different Angles 

**Figure 3:**
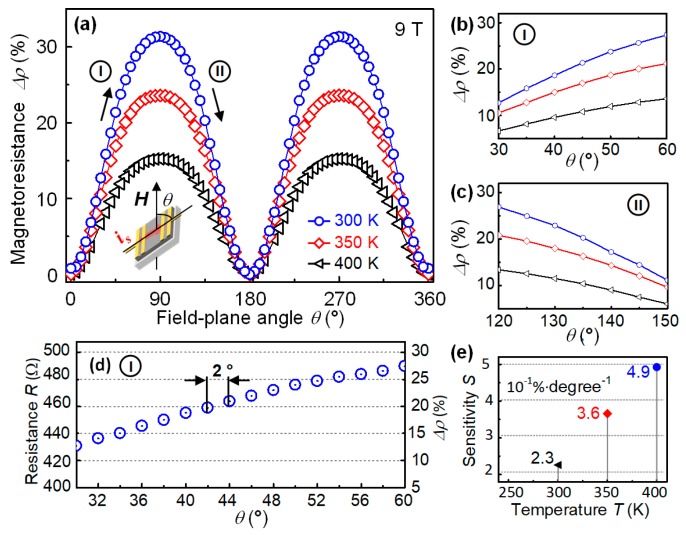
Magnetic sensing property of carbon film in external magnetic field with different plane-to-field angle *θ*. (**a**) Temperature dependent anisotropy magnetoresistance from 300–400 K. The intensity of the external field was 9 T, *θ* changed from 0° to 360° as sample rotated along axis perpendicular to magnetic line and current direction, as illustrated inside the figure; (**b**,**c**) near-linear AMR response in the range *θ* of 30°–60°, and the temperature dependency; (**d**) resistance and magnetoresistance in the angle range of 30°–60° at 300 K, with increment of 2°, implying that the finest resolution can reach 2°; (**e**) calculated sensitivity at different temperatures from (**b**,**c**).

Another common application for magnetic sensors is to detect the directional change of the magnetic lines, which can be used for providing the rotating speed, angle of certain motive part in a mechatronic system such as electric motors. Aiming at this potential utilization, the anisotropic magnetoresistance (AMR) of the sensing device was investigated on a rotating holder in the external field of 9 T and temperature range of 300–400 K. The initial position of the device is not parallel to the magnetic field, at which the relative angle of film surface and magnetic line *θ* is 0°. The testing source current *i_s_* is along the initial magnetic field direction. When AMR measurement started, the rotating axis of the device was perpendicular to the magnetic field and *i_s_*, as illustrated in Figure 3a. The AMR performance as *θ* increased from 0° to 360° is shown in Figure 3a. The results clearly showed a cosine-like periodic change in spite of the temperature difference. The resistance of the device reached maximum value at 90° and 270°, at which the surface plane of the device is normal to the magnetic field. In this position, the magnetic line is normal to *i_s_*. Such configuration is opposite to that of the few-layer graphene device [24], which showed maximum resistance when the magnetic field is parallel to *i_s_*. In contrary, the minimum resistance was achieved at 0°, 180°, and 360°, where the device surface is perpendicular to the magnetic field, that is, the magnetic line parallel to *i_s_*. Moreover, the graphene device showed minimum resistance when the magnetic line being normal to *i_s_*. The above difference between nano-crystallited carbon film and graphene may come from the vertical orientation of the graphene layers in the nano-crystallited film. According to previous studies, the magnetic sensing property of graphene-based device is optimized when *i_s_* is normal to the graphene plane. Owing to the particular orientation of the nano-crystallites in our film, the optimized *i_s_* direction is exactly in the plane of the film surface, not the through-film direction in previous graphene-based device. This feature gives our device a huge advantage considering application, because it is much easier to build all electrodes just on the top surface of the film than on the bottom and top of few-atomic-layers of graphene. It also can be seen that the AMR changing ratio decreased as temperature increased from 300 to 400 K. This may be due to the increasing thermal fluctuation in the graphene nano-crystallites, leading to less ordered spin alignment and weaker intrinsic magnetism [22]. Therefore, the magnetic field has less influence in the transport of the carriers.

Figure 3b,c shows the near-linear responding regions of the device, which are 30°–60°(I) and 120° to 150°(II), respectively. Due to the periodic performance of the device, other two near-linear regions can also be expected in 210°–240° and 300°–330°. In order to estimate the best sensing ability of the device to minor rotating angle change, we chose the near-linear responding region at high temperature for evaluation, which was 30°–60° at 400 K, with the increasing step of 2°. Figure 3d exhibits the plots of the absolute resistance value of the device in each step of increasing field intensity and their corresponding MR value. Apparently, the reading of the data showed a monotonic increase as *θ* increased, and the increment between each step was about 4 Ω, which was easy to detect for the measurement equipment. The fluctuation of the data plots was less than that in Figure 2d, because the measurement was carried out in static magnetic field. It can be inferred form the Figure 3d that at current circumstance, the best angular resolution of the device can reach to 2°. Figure 3e summarizes the angular sensitivity of the device in the near-linear responding region in different temperatures. As the temperature increased from 300 to 400 K, the sensitivity increased from 0.23% to 0.49% per degree. This also indicated that the temperature should be calibrated if the device is used for detecting the direction of magnetic field or relative angle of the rotating parts. However, if the device was used for detecting rotating speed, such calibration will be unnecessary.

### 3.4. The Enhancement of MR Performance by n-Si Substrate

**Figure 4:**
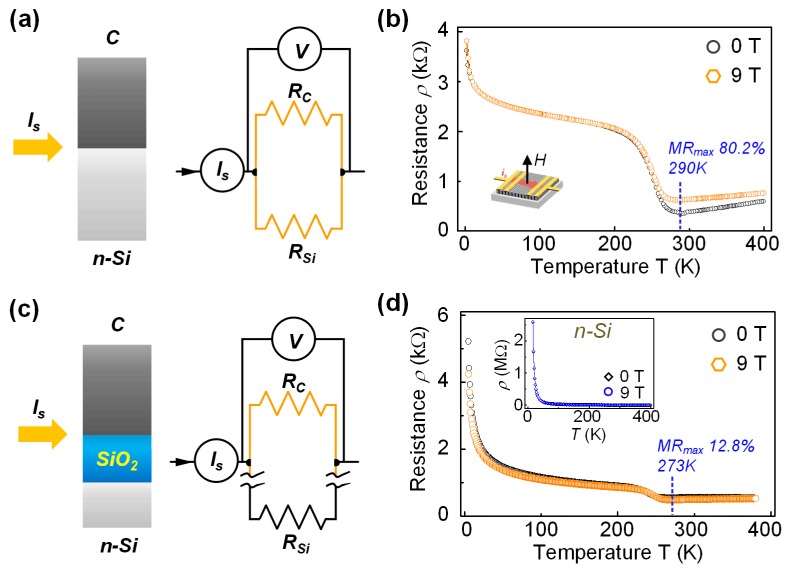
(**a**) Schematic illustration and equivalent circuit of the C-film/n-Si device. *I_s_* is the source current, and *R_c_* and *R_si_* represent the resistance of the carbon film and n-Si substrate, respectively. (**b**) R–T relationship of the C-film/n-Si device in 0 T and 9 T conditions. (**c**) Schematic illustration and equivalent circuit of the C-film/SiO_2_ device. (**d**) R–T relationship of the C-film/SiO_2_ device in 0 T and 9 T conditions. Inset figure is the R–T relationship of n-Si substrate.

Now, we would like to discuss the influence of the substrate in our device. In this study, the nano-crystallited carbon film was deposited on n type silicon substrate. The oxide layer on the n-Si surface was removed by ion plasma cleaning before deposition, which was confirmed by side-view TEM observation (see Figure 1a). Therefore, the film and substrate were well contacted and can be considered as two parallel connected resistors, and *i_s_* will flow through the film *R_c_* and substrate *R_si_*, as illustrated in Figure 4a. Figure 4b shows the temperature dependence of the resistance of the n-Si/ device. For comparison, we introduced another n-Si substrate with 300 nm SiO_2_ as a dielectric layer, deposited the nano-crystallited carbon film and built a device with the same wiring configuration. The dielectric layer cut off the electric conduction in the n-Si substrate, thus, *i_s_* only flowed through *R_c_*, as illustrated in Figure 4c. The temperature dependences of the resistance of the devices were measured in 0 T and 9 T-field conditions, and the results are exhibited in Figure 4b and d. Moreover, the inset Figure 4d also exhibited the R–T relationship of n-Si substrate. According to the results, the R–T relationship of C-film/n-Si device was similar to that of C-film itself, but different from that of n-Si. This phenomenon implied that the carbon film in the n-Si/C device played a dominant role in the electric conduction process. Moreover, the largest MR ratio of the C-film/n-Si device was at 290 K, close to that of C-film, 273 K. The most sensitive region of the two devices are both around 300 K. On the other hand, n-Si did not show such feature, with the largest MR value at room temperature less than 1%. Therefore, it can be inferred that the MR behavior we found in this study is mainly from carbon film, rather than from silicon substrate. 

The measured results in Figure 4b,d were the overall resistance of the device *R_d_*. For C-film/n-Si device, *R_d_* has the relationship with *R_c_* and *R_si_* as 1/*R_d_* = 1/*R_c_* + 1/*R_si_*, and since *R_si_* was also measured, *R_c_* can be calculated. For C-film/SiO_2_ device, *R_d_* equaled to *R_c_*. Consuming the resistance of n-Si substrate increased by 1%, the resistance of n-Si at 9 T was calculated. Furtherly, the MR value of C-film was obtained by comparing the calculated resistance in 0 T and 9 T conditions, as listed in Table 1. The ideal value of carbon magnetoresistance can reach to 10,711.28% in the magnetic field of 9 T according to the table, which is approximate 1190% per T. We also checked the resistivity of the n-Si substrate with calculation based on the size, electrode-distance, and thickness of the substrate. The calculated resistivity was 25.04 Ω·cm, which coincided with the given information from the supplier (20–30 Ω·cm).

The large MR value of carbon film is close to the magnetoresistance of few-layer graphene device in certain configuration [25], in which the scattering of carrier electrons between graphene and metal interface by Lorenz force. It should be noted that the MR performance of the carbon film was remarkably improved by using n-Si as the substrate instead of SiO_2_/n-Si. The SiO_2_ layer is insulating, which can isolate the carbon film from n-Si during electron transport, so as the n-Si does not influence the MR behavior of the carbon film. Therefore, the MR performance only comes from the magnetism of pure C-film. On the other hand, when the C-film was grown directly on n-Si, they could share the itinerary electrons which mainly comes from the n-Si, considering the n-Si volume was much larger than C-film volume (their thicknesses are 500 μm and 35 nm). Thus, the n-Si worked as an electron donor to the C-film, which provided itinerary electrons to C-film. The magnetism in the C-film is originated from the ferromagnetic spin-ordering at the edge of graphene layers, and such ordering can be enhanced by increasing itinerary concentration [22]. Therefore, with larger itinerary electron concentration, the magnetism of the C-film was greatly enhanced, and its MR behavior was exceedingly improved. However, due to the low MR property of Si itself, the substrate inevitably hindered the sensing performance of the device. In future studies, if the n-Si substrate could be substituted with a more effective manner, a great leap of magnetic sensing ability can be expected on carbon-film-based devices. Thus, the realization of a commercial carbon-based film magnetic sensor can be expected.

## 4. Conclusions

In this study, n-type silicon was introduced as a “donor” substrate to improve the MR performance of graphene nano-crystallited carbon films. The magnetic sensing performances of the n-Si/C-film were investigated in the temperature range of 250–400 K, and the optimized temperature of the magnetic sensing performance was found to be 300 K. The linear responding of the device to increasing intensity of external magnetic field was found in strong field range (3–9 T), and the intensity-increment of 1 mT was recognized in the best-resolution region. AMR test showed a cosine-like periodic responding feature, and the angle-change of 2° was identified in the near-linear responding region. The R–T curves of the C-film/n-Si and C-film/SiO_2_ devices exhibited similar temperature dependency, and their largest MR values appeared both around 300 K, indicating that the magnetic sensing property was from nano-crystallited carbon film. The enhancement of n-Si substrate on the magnetic sensing ability was discussed, and by calculating the contributions of n-Si substrate and carbon film on the overall MR of the device, the enhanced MR of carbon film could reach to over 1100%/T, which was close to graphene/metal device. This research provided a simple, effective way to improve the MR performances of nano-crystallited carbon film and predicted a great leap of the magnetic sensing property of the carbon-based device by pointing out that the n-Si enhancement being substituted with other manners.

## Figures and Tables

**Table 1:** The resistances and magnetoresistance (MR) values of the film and substrate in the C-film/n-Si device.

	0 T	9 T	MR
C-film/n-Si device *R_d_*	380.09	684.92	80.1%
n-Si substrate *R_si_*	683.15	689.83	1%
C-film (calculated) *R_c_*	856.79	92630	10711.28%

## References

[1] Xue P., Chen C., Diao D. (2019). Highly sensitive flexible strain sensor based on wribkled graphene nanocrystallite carbon film. Carbon.

[2] Zhai W., Srikanth N., Kong L., Zhou K. (2017). Carbon nanomaterials in tribology. Carbon.

[3] Kim S., Park S., Park H., Park D., Jeong Y., Kim D. (2017). Highly sensitive and multimodal all-carbon skin sensors capable of simultaneously detecting tactile and biological stimuli. Adv. Mater..

[4] Adhikari B.-R., Govindhan M., Chen A. (2015). Carbon Nanomaterials Based Electrochemical Sensors/Biosensors for the Sensitive Detection of Pharmaceutical and Biological Compounds. Sensors.

[5] Wang Z.M., Zhang J., Han X., Li Q.F., Wang Z.L., Wei R. (2014). Corrosion and salt scale resistance of multilayered diamond-like carbon film in CO2 saturated solutions. Corros. Sci..

[6] Wang F., Liu S., Shu L., Tao X.-M. (2017). Low-dimensional carbon based sensors and sensing network for wearable health and environmental monitoring. Carbon.

[7] Yang W., Ratinac K.R., Ringer S.P., Thordarson P., Gooding J., Braet F. (2010). Carbon nanomaterials in biosensors: Should you use nanotubes or graphene?. Angew. Chem. Int. Ed..

[8] Vaseashta A., Dimova-Malinovska D. (2005). Nanostructured and nanoscale devices, sensors and detectors. Sci. Technol. Adv. Mater..

[9] Wanekaya A.K. (2011). Applications of nanoscale carbon-based materials in heavy metal sensing and detection. Anal..

[10] Obitayo W., Liu T. (2012). A Review: Carbon Nanotube-Based Piezoresistive Strain Sensors. J. Sens..

[11] Lipomi D.J., Vosgueritchian M., Tee B.C.-K., Hellstrom S.L., Lee J.A., Fox C.H., Bao Z. (2011). Skin-like pressure and strain sensors based on transparent elastic films of carbon nanotubes. Nat. Nanotechnol..

[12] Wei N., Huang H., Liu Y., Yang L., Wang F., Xie H., Zhang Y., Wei F., Wang S., Peng L. (2016). Nanoscale color sensors made on semiconducting multi-wall carbon nanotubes. Nano Res..

[13] El-Kady M.F., Shao Y., Kaner R.B. (2016). Graphene for batteries, supercapacitors and beyond. Nat. Rev. Mater..

[14] Colombo L., Wallace R.M., Ruoff R.S. (2013). Graphene Growth and Device Integration. IEEE Proc..

[15] Kataria S., Wagner S., Ruhkopf J., Gahoi A., Pandey H., Bornemann R., Vaziri S., Smith A.D., Östling M., Lemme M.C. (2014). Chemical vapor deposited graphene: From synthesis to applications. Phys. Status Solidi A.

[16] Wang C., Diao D., Fan X., Chen C. (2012). Graphene sheets embedded carbon film prepared by electron irradiation in electron cyclotron resonance plasma. Appl. Phys. Lett..

[17] Wang C., Chen C., Diao D. (2016). Top surface modification of carbon film on its structure, morphology and electrical resistivity using electron-ion hybrid irradiation in ECR plasma. Surf. Coat. Technol..

[18] Wang C., Diao D. (2013). Magnetic behavior of graphene sheet embedded carbon film originated from graphene crystallite. Appl. Phys. Lett..

[19] Wang C., Zhang X., Diao D. (2015). Nanosized graphene crystallite induced strong magnetism in pure carbon films. Nanoscale.

[20] Chen C., Xue P., Fan X., Wang C., Diao D. (2018). Friction-induced rapid restructuring of graphene nanocrystallite cap layer at sliding surfaces: Short run-in period. Carbon.

[21] Wang P., Diao D. (2017). Low friction of graphene nanocrystalline embedded carbon nitride coatings prepared with MCECR plasma sputtering. Surf. Coat. Technol..

[22] Wang C., Diao D. (2017). Self-magnetism induced large magnetoresistance at room temperature region in graphene nanocrystallited carbon film. Carbon.

[23] Wang C., Diao D. (2011). Cross-linked graphene layer embedded carbon film prepared using electron irradiation in ECR plasma sputtering. Surf. Coatings Technol..

[24] Liao Z.-M., Kumar S., Duesberg G.S., Cross G.L.W., Shvets I.V., Wu H.-C., Wu H.-C., Zhou Y.-B., Yu D.-P., Wu H. (2012). Large Magnetoresistance in Few Layer Graphene Stacks with Current Perpendicular to Plane Geometry. Adv. Mater..

[25] Lu J., Zhang H., Shi W., Wang Z., Zheng Y., Zhang T., Wang N., Tang Z., Sheng P. (2011). Graphene magnetoresistance device in van der Pauw geometry. Nano Lett..

